# Transcriptional Regulation of the *rsbV* Promoter Controlling Stress Responses to Ethanol, Carbon Limitation, and Phosphorous Limitation in *Bacillus subtilis*

**DOI:** 10.1155/2010/263410

**Published:** 2010-05-03

**Authors:** Soo-Keun Choi, Milton H. Saier

**Affiliations:** ^1^Division of Biological Sciences, University of California at San Diego, La Jolla, CA 92093-0116, USA; ^2^Korea Research Institute of Bioscience & Biotechnology (KRIBB), 52 Oun-Dong, Yusong, Taejon 305-333, South Korea

## Abstract

The *σ* ^B^-dependent promoter in front of the *rsbV* gene of *Bacillus subtilis* is induced ∼5-fold in response to (1) the addition of 4% ethanol, (2) carbon starvation, and (3) phosphorous starvation. Binding sites for the global carbon and nitrogen regulators, CcpA and TnrA, were mutated, and the consequences of their loss and that of CcpA or TnrA were studied using *rsbV-lacZ* fusions. These responses proved to be dependent on CcpA, TnrA, and their putative binding sites upstream of the promoter. Induction in response to glucose limitation was largely abolished by loss of CcpA or the upstream region, while induction in response to phosphorous limitation was largely abolished only by the upstream mutations. The results suggest that CcpA directly influences the carbon starvation response and that both proteins exert indirect effects on all three stress responses. The integrity of the DNA sequence is important for all three responses.

## 1. Introduction

In Gram-positive bacteria such as species of *Bacillus*, the complex *rsb* operon encodes the stress sigma factor, *σ* ^B^, and many of its regulatory proteins [1, 2]. This operon includes two promoters, a *σ* ^A^-dependent promoter in front of the *rsbR* gene at the beginning of the operon, and a *σ* ^B^-dependent promoter in front of the *rsbV* gene, the fifth gene in this 8-cistron operon [3]. Much is known about the biochemical mechanism of sigma B control which involves an anti-*σ* ^B^, an anti-anti-*σ* ^B^, and protein phosphorylation [4–6]. Additionally, induction of the *σ* ^B^promoter in part mediates adaptation to heat shock, (40–54°C), cold shock (4–12°C), NaCl (10%) shock, ethanol (4–10%) shock, and various starvation (carbon, phosphorous, and nitrogen) stresses [5, 7]. It has been argued that a drop in ATP levels may trigger *σ* ^B^ activation [8]. Elements of this regulatory system are found in a wide range of bacteria which in addition to low and high G + C, Gram-positive bacteria include bacteroidetes, cyanobacteria, proteobacteria, and deinococci [2]. 

We have investigated the dependencies of various stress responses (ethanol stress and limitation for carbon or phosphorous), showing that these responses are dependent on the principal carbon-limitation transcription factor in *B. subtilis*, CcpA [9–11], as well as the principal nitrogen regulator TnrA [12]. However, we show that point mutations and deletions in the region upstream of the *rsbV* promoter, within the *rsbU* gene, largely abolish all of these stress responses. Because these studies were conducted with an *rsbV-lacZ* fusion reporter system integrated at the *amyE* locus, we can conclude that RsbU function is not involved in these effects. Our preliminary results suggest that CcpA and TnrA exert their effects (with the possible exception of carbon starvation) through secondary effects. They further suggest that the DNA conformation upstream of the *rsbV* promoter plays a critical role in responses of the *rsbV* promoter to multiple stresses.

## 2. Materials and Methods

### 2.1. Strains and Plasmids

Bacterial strains used in this study are listed in Table 1. *E. coli* DH5*α* was used as a general cloning host. Transformation of *B. subtilis *was carried out by a method described elsewhere [13]. Plasmid pDG1661 was obtained from the *Bacillus *Genetics Stock Center in Ohio. The promoter of *rsbV* was amplified by PCR using Platinum Pfx DNA polymerase (Invitrogen Corp.) with primers rsbV1 (5′-ataaagcttttctcggcatctcgcaggac-3′) and rsbV2 (5′-aaaggatcccaagcctgtaatgtcaaaca-3′), digested with *Hin*dIII and *Bam*HI and cloned into the corresponding sites of plasmid pDG1661 to construct pDG-*rsbV1*. The fragment *rsbV3* amplified by PCR with primers rsbV7 (5′-tataagcttgaatgcagaacggaaaacgg-3′) and rsbV2 was digested with *Hin*dIII and *Bam*HI and cloned into the corresponding sites of plasmid pDG1661 to construct pDG-*rsbV3*. The fragment *rsbV5* amplified by PCR with primers rsbV11 (5′-tataagcttcttggagcgtcctgatctgc-3′) and rsbV2 was digested with *Hin*dIII and *Bam*HI and cloned into the corresponding sites of plasmid pDG1661 to construct pDG-*rsbV5*. The promoter of *ctc* was amplified by PCR with primers ctc1 (5′-ttgaattctgaatattgtaggtaacatc-3′) and ctc2 (5′-ttaggatccatttttgccttcgtccctc-3′), digested with *Eco*RI and *Bam*HI, and cloned into the corresponding sites of plasmid pDG1661 to construct pDG-*ctc*.

### 2.2. Site-Directed Mutagenesis

The *Sal*I fragment containing the *rsbV* promoter, TRE (the TnrA binding site), and CRE (the CcpA binding site) from pDG-*rsbV1* were cloned into pUC19 to construct pUC-*rsbV1*. pUC-*rsbV1m1* containing the mutated CRE site in which the central C was changed to A was constructed by site-directed mutagenesis of pUC-*rsbV1* using a QuikChange Site-Directed Mutagenesis kit (Stratagene Corp.) with primers rsbV5 (5′- gaatgcagaacggaaaaagctttcttggagcgtcctg-3′) and rsbV6 (5′-caggacgctccaagaaagctttttccgttctgcattc-3′). The mutation was confirmed by sequencing. The *Eco*RI and *Bam*HI fragment of pUC-*rsbV1m1* containing the *rsbV* promoter and the mutated CRE was cloned into pDG1661 to construct pDG-*rsbV1m1*.

### 2.3. Growth Conditions and Enzyme Assays

*Bacillus* cells were grown in LB, glucose limitation medium (GLM), and phosphate limitation medium (PLM) [14]. GLM contains 0.05% glucose and the 0.15 mM of KH_2_PO_4_ was used for PLM. But the similar results was obtained from 0.18 mM of KH_2_PO_4_. Environmental stress was imposed by adding ethanol to a final concentration of 4% (vol/vol) to cells growing exponentially in LB medium. For the *β*-galactosidase assay, overnight cultures grown in 1 mL of corresponding medium were diluted 200-fold into fresh medium and grown at 37°C with shaking at 200 rpm. Samples were collected at hourly intervals for the determination of the optical density at 600 nm. *β*-Galactosidase activity was determined as previously described [15].

## 3. Results

### 3.1. Strain Construction

**Figure 1:**
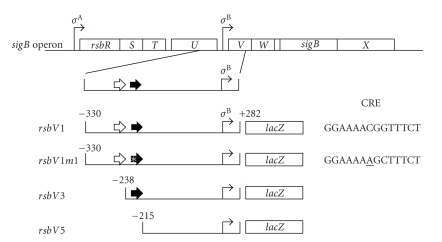
Schematic depiction of the *rsb* or *sigB* operon in *B. subtilis* (top) as well as the constructs made in order to determine the involvement of putative CcpA and TnrA binding sites in control of *σ* ^B^ promoter activity in response to various stresses. The octacistronic operon as well as the two promoters, one dependent on *σ* ^A^-RNA polymerase in front of the *rsbR* gene and one dependent on *σ* ^B^-RNA polymerase in front of the *rsbV* gene, are shown. The wild-type control region (nucleotides −330 to +282) including the putative TnrA binding site (open arrow), the putative CcpA binding site (closed arrow), and the *σ* ^B^ promoter was fused to the *lacZ* gene encoding *β*-galactosidase as the reporter gene. The wild-type construct is designated by *rsbV1*. In the second construct, *rsbV1m1*, the CcpA binding site (CRE) was mutated, changing the central conserved C to an A (see sequence on right-hand side of the construct). In the *rsbV3* construct, the control region started at position −238, and the TnrA binding site (TRE) was thereby deleted. In construct *rsbV5*, the control region started at position −215, and consequently the CRE site as well as the TnrA binding site was thereby deleted. All constructs were inserted in the chromosome at the *amyE* locus.

Figure 1 shows the constructs made and the mutations introduced in order to investigate the ethanol stress, carbon limitation, and phosphorous limitation responses of the *σ* ^B^-dependent promoter in the *sigB* (*rsb*) operon. The *rsb* operon has a stress-insensitive *σ* ^A^ promoter in front of the *rsbR* gene, indicated by the first arrow above the operon, and a stress-sensitive *σ* ^B^ promoter in front of the *rsbV* gene, indicated by the second arrow above the operon [3]. Upstreams of the *σ* ^B^ promoter and in the *rsbU* structural gene are two putative transcription factor-binding sites, one specific for TnrA (open arrow) and one specific for CcpA (a so-called catabolite responsive element (CRE) (closed arrow) see Figure 1) [16]. A *lacZ* fusion was constructed at position +282, well after the *σ* ^B^ promoter. The different strains have (1) the wild-type regulatory region (*rsbV1*), (2) the same with a C → A point mutation in the CcpA binding site (*rsbV1m1*), (3) a deletion lacking the putative TnrA binding site (*rsbV3*), and (4) a deletion lacking both the TnrA and CcpA binding sites (*rsbV5*) (Figure 1). Mutation of the conserved C in the CRE is expected to abolish CcpA binding [17].

### 3.2. The Ethanol Stress Response

**Figure 2:**
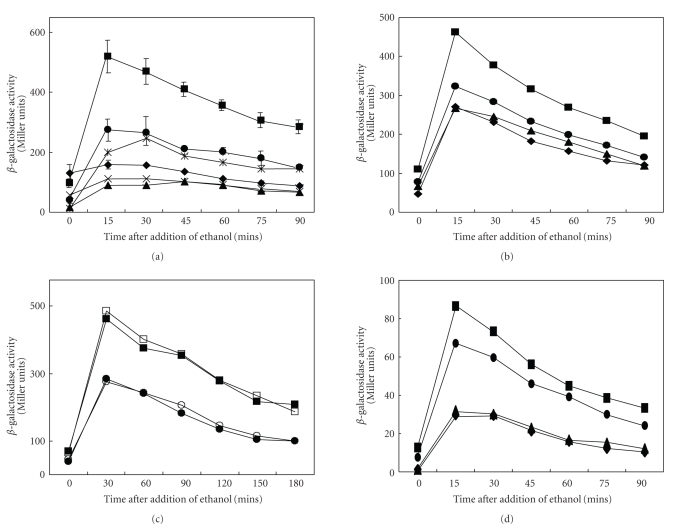
Effects of various mutations and conditions on the ethanol stress response of the *σ* ^B^-dependent *rsbV* promoter with cells growing exponentially in LB medium. (a) At time = 0, ethanol was added to a final volume of 4%, giving rise to a 4-5-fold enhancement of promoter activity in the wild-type genetic background (*rsbV1*) (■). The mutations (Figure 1) decreased the basal as well as the induced activities of the promoter. Wild type (■); *ccpA* mutant (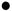); *hprK* mutant (×); the *rsbV1m1* mutant (*◆*); *rsbV3* (lacking the TnrA binding site) (▾) *rsbV5* (lacking the TnrA and CcpA binding sites) (▴). (b) Effects of the loss of CcpA (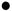), TnrA (▴), or both transcription factors (*◆*) on the ethanol stress response of the *σ* ^B^-dependent promoter in the *rsb* operon. (c) Effect of glucose on the ethanol stress response of the *rsbV* promoter. The experiment was conducted in LB medium with (closed symbols) and without (open symbols) 1% glucose. The wild type (squares) and *ccpA* mutant (circles) were examined. (d) Response of the *σ* ^B^-dependent *ctc* gene to ethanol stress. A *lacZ* fusion was made to the *ctc* gene, and the response to 4% ethanol was measured in the wild-type background (■), the *ccpA* mutant background (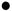), the *rsbV1m1* genetic background (*◆*), and the *rsbV5* genetic background (▴). The experiment was conducted in LB medium under standard conditions. Ethanol (4%) was added at *t* = 0.

The results of studies on the ethanol stress response with these constructs are presented in Figures 2(a)–2(d). Upon addition of 4% ethanol, *σ* ^B^-promoter activity increased about five fold in the wild-type genetic background in agreement with earlier studies [5, 18]. As shown in Figures 2(a) and 2(b), the loss of either CcpA or HprK, both required for catabolite repression in *B. subtilis*, resulted in a moderate loss of the ethanol activation response. The loss of TnrA or of both TnrA and CcpA resulted in slightly less response than when just the *ccpA* gene was deleted. 

Figure 2(a) shows that the loss of the upstream regulatory region including the TnrA site (*rsbV3*), the loss of both the TnrA and CcpA binding sites (*rsbV5*), or the point mutation in the CcpA binding site (*rsbV1m1*) resulted in a much more substantial loss of activation. The three mutant constructs showed comparable responses. These results show that while CcpA and TnrA may play regulatory roles, a point mutation in the CcpA binding site or modification of the upstream region with loss of the TnrA binding site either alone or together with the CRE has a much more dramatic effect. 

We then examined the effect of glucose on the ethanol response (Figure 2(c)), both in the wild-type genetic background (squares) and in the *ccpA* mutant background (circles). With glucose (closed symbols), the ethanol response was identical to that without glucose (open symbols) within experimental error. We therefore conclude (1) that the DNA sequence in front of the *σ* ^B^ promoter is more important to regulation than the presence or absence of CcpA and/or TnrA, and (2) that glucose does not influence the ethanol stress response either with or without CcpA [19]. Since the action of CcpA usually depends on the presence of glucose [20], we suggest that CcpA and TnrA exert indirect effects on the activation of the *σ* ^B^ promoter in response to ethanol addition (see Section 4). The fact that even a single nucleotide substitution in the CRE has as dramatic an effect as deletion of the entire region suggests (but does not prove) that the CRE is important for the ethanol stress response even though CcpA and glucose are not. If this suggestion is correct, then a requirement for the presence of the TnrA binding site can also be inferred (see Section 4). 

Figure 2(d) shows the effects of various mutations on the ethanol stress response of the *σ* ^B^-dependent *ctc* gene in *B. subtilis*. Constructs were made and inserted at the *amyE* locus as described in “Section 2.” The experiment was conducted in LB medium with the addition of 4% ethanol at *t* = 0. The responses observed followed those for the *rsb* operon. Thus, the *ccpA* mutation moderately diminished the activity of the *ctc* promoter, relative to that of the wild type, but the *rsbV1m1* and *rsbV5* mutations exerted much more drastic effects. On the basis of these results, we suggest that the effects observed on the stress response of *rsbV* operon expression are transmitted to the *ctc* gene, and that *σ* ^B^ is therefore rate limiting for expression of the *ctc* gene. These results also confirm the validity of the data presented in Figure 2(a).

### 3.3. The Carbon Limitation Response

**Figure 3:**
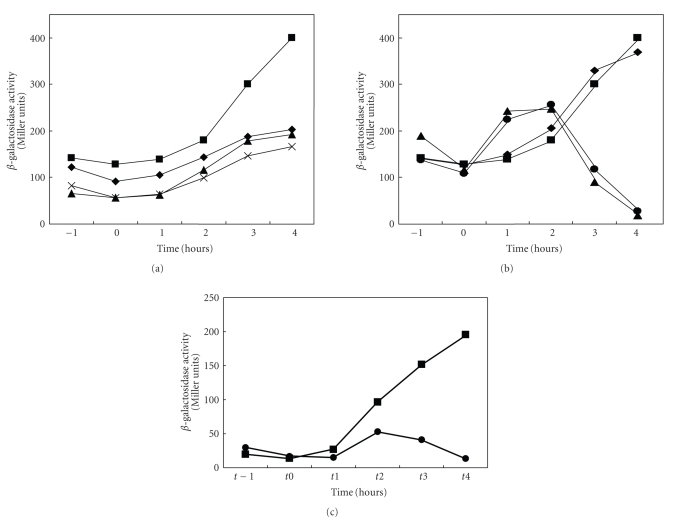
Effects of various mutations and conditions on the glucose starvation response of the *rsbV* promoter. (a) Effects of the *rsbV1m1* (*◆*), *rsbV3* (▴), and *rsbV5* (×) mutations in the *rsbU* (upstream) region in front of the *rsbV* promoter on promoter activity in response to glucose limitation as a function of time. The wild-type response (*rsbV1*, ■) is shown at the top. (b) The effects of *ccpA* (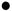), *tnrA* (*◆*), and *ccpA tnrA* (▴) double knockout mutants on the glucose starvation response as a function of time. The wild-type response is represented by square symbols (■). (c) Effect of glucose limitation on expression of the *ctc* gene which is under sigma B (*σ* ^B^) control, in the wild-type (■) or *ccpA* (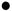) genetic backgrounds.

As observed upon the addition of ethanol, glucose limitation causes a substantial increase in expression of the *rsbV* promoter in a wild-type genetic background (Figure 3). The response to a point mutation or loss of part or most of the upstream region in the *rsbU* gene was dramatic. The basal activity was diminished only slightly by the point mutation, but starvation induction was largely abolished. The deletion mutations reduced the basal activity more, but in all cases, an inductive response, although much reduced, was still observed (Figure 3(a)). 

Figure 3(b) shows the effects of loss of TnrA, CcpA, or both factors. Loss of TnrA was essentially without effect, but the loss of CcpA ± TnrA resulted in premature induction followed by dramatic repression. When the consequences of the loss of CcpA were determined for the *σ* ^B^-controlled *ctc* gene (Figure 3(c)), the dramatic induction observed upon glucose starvation was essentially abolished. The relative responses of the *rsbV* and *ctc* promoters were comparable.

### 3.4. The Phosphorous Limitation Response

**Figure 4:**
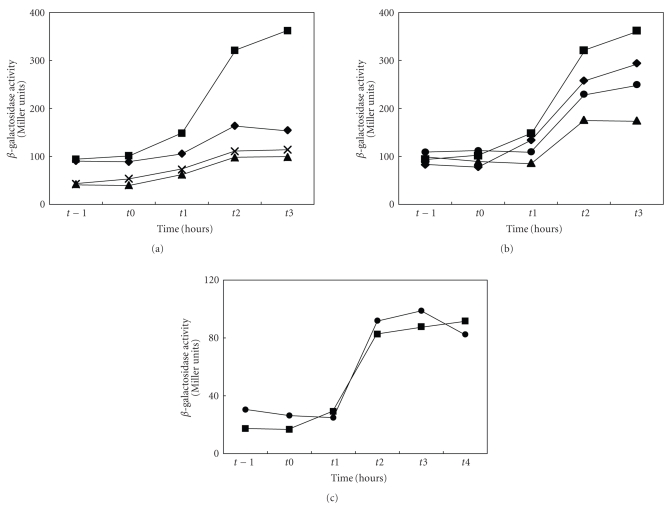
Effects of various mutations and conditions on the phosphorous starvation response. (a) Wild-type response (*rsbV1*, ■) to phosphate limitation, and the effects of the *rsbV1m1* (*◆*), *rsbV3* (▴), and *rsbV5* (×) mutations on this response as a function of time. (b) Effects of the loss of CcpA (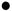), TnrA (*◆*), and both transcription factors (▴) as compared with wild type (■) on the phosphate limitation response. (c) Lack of effect of the loss of CcpA (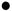) on the phosphate limitation response of the *ctc* gene which is under sigma B (*σ* ^B^) control. The wild-type response is represented by ■.

Phosphorous limitation similarly caused substantial induction of the *rsbV* promoter (Figure 4(a)). Mutation of the CRE or deletion of part or most of the upstream region greatly reduced this response. The effects of loss of CcpA and/or TnrA proved to be less dramatic with the loss of CcpA having a greater effect than the loss of TnrA (Figure 4(b)). Loss of both reduced expression even further. Correlating with the minimal response to the loss of CcpA on *rsbV *promoter expression, induction of expression of the *ctc* gene was not appreciably altered by deletion of the *ccpA* gene (Figure 4(c)).

## 4. Discussion

At the onset of this project, we discovered binding sites for two transcription factors in front of the stress-responsive *rsbV* promoter within the *rsbU* structural gene. The first was a typical binding site for the global nitrogen regulator of *B. subtilis*, TnrA, while the second one was a typical CRE site which normally binds the global carbon regulator of *B. subtilis*, CcpA. We introduced a debilitating point mutation in the CRE and deleted either the TnrA binding site or both the TnrA and CcpA binding sites (see Figure 1). Surprisingly, regardless of the type of mutation introduced, changing this upstream region largely abolished the stress responses to (1) ethanol addition, (2) carbon limitation, and (3) phosphorous limitation. In all cases, the responses were reduced to similar degrees, and all three types of mutations had similar, but not identical, effects. 

Multiple interpretations are possible for these observations. For example, the CRE and adjacent regions might bind a protein or RNA, and binding might depend on the secondary structure of the upstream DNA. Even subtle changes introduced by a point mutation in the CRE might abolish the activating interactions. Alternatively, the upstream region might inherently control promoter activity in response to stress signals by transmitting a signal through the DNA helix to the promoter. This could affect binding of RNA polymerase or another factor that contributes to the stress responses. Still, another possibility is that the CRE and the TnrA binding sites are important to the stress induction, but they do not function primarily in this regard to respectively bind CcpA and TnrA. Regardless, our experiments clearly indicate that the generalized effects of these upstream changes in the DNA are not mediated by either CcpA and/or TnrA alone. 

We believe that these transcription factors, CcpA and TnrA, exert their effects which in general are less dramatic than those of the upstream mutations, through indirect means. For example, the responses to ethanol and phosphorous limitation stresses were only moderately dependent on CcpA and TnrA. Further, although CcpA binding to the DNA is usually dependent on the presence of metabolites generated in the presence of exogenous glucose or another glycolytically metabolized sugar, the presence of glucose exerted no effect on the ethanol stress response (Figure 2(c)). By contrast, carbon starvation induction of the *rsbV* promoter was dramatically affected by the loss of CcpA although deletion of *tnrA* was without effect. Since the *ccpA* mutation affects growth rate and glucose utilization, it is possible that even this dramatic effect of CcpA could be indirect. Regardless, its effect does not appear to be mediated by the CRE identified in the *rsbU* gene. 

The studies reported here clearly indicate the importance of the *rsbU* region upstream of the *rsbV* promoter on both basal and induced promoter activities. It is clear, also, that both TnrA and CcpA influence these stress responses. The phenomenology is therefore defined. The detailed molecular mechanisms, providing explanations for the observations reported here, have yet to be determined. The functions of the putative binding sites for TnrA and CcpA in the *rsbU* gene similarly must be investigated further.

## Figures and Tables

**Table 1:** *Bacillus subtilis *strains used in this study.

Strain	Relevant genotype	Source or reference^(a)^
JH642	*trpC2 pheA1*	BGSC
ST124	*trpC2 pheA1 ccpA*::*Tn917 *	[2]
ST106	*trpC2 pheA1 *Δ*(bgalX) amyE::(gntRK*′*-lacZ) *Δ*spo0E ptsK::Spc*	Laboratory stock
SF416T	*trpC2 amyE::(amtB-lacZ)416 neo tnrA62::Tn917erm*	Susan Fisher
BCS50	*trpC2 pheA1 amyE::rsbV1-lacZ*	pDG-*rsbV1* tf JH642
BCS52	*trpC2 pheA1 amyE::rsbV1-lacZ ccpA::Tn917*	ST124 tf BCS50
BCS54	*trpC2 pheA1 amyE::rsbV1m1-lacZ*	pDG-*rsbV1m1* tf JH642
BCS55	*trpC2 pheA1 amyE::rsbV3-lacZ*	pDG-*rsbV3* tf JH642
BCS56	*trpC2 pheA1 amyE::rsbV5-lacZ*	pDG-*rsbV5* tf JH642
BCS58	*trpC2 pheA1 amyE::rsbV1-lacZ ptsK::Spc*	ST106 tf BCS50
BCS61	*trpC2 pheA1 amyE::ctc-lacZ*	pDG-*ctc* tf JH642
BCS62	*trpC2 pheA1 amyE::ctc-lacZ ccpA::Tn917*	ST124 tf BCS61
BCS68	*trpC2 pheA1 amyE::rsbV1-lacZ tnrA62::Tn917erm*	SF416T tf BCS50
BCS69	*trpC2 pheA1 amyE::rsbV1-lacZ tnrA62::Tn917erm ccpA::Tn917*	ST124 tf BCS68

^(a)^tf, the indicated chromosomal DNA or plasmid was used to transform the indicated recipient strain.
